# Biased TCR gene usage in citrullinated Tenascin C specific T-cells in rheumatoid arthritis

**DOI:** 10.1038/s41598-021-04291-8

**Published:** 2021-12-31

**Authors:** Ravi K. Sharma, Sanjay V. Boddul, Niyaz Yoosuf, Sara Turcinov, Anatoly Dubnovitsky, Genadiy Kozhukh, Fredrik Wermeling, William W. Kwok, Lars Klareskog, Vivianne Malmström

**Affiliations:** 1 Division of Rheumatology, Department of Medicine, Rheumatology Unit, Karolinska Institutet, Karolinska University Hospital, 171 76 Stockholm, Sweden; 2 Center for Molecular Medicine, Karolinska Institutet, Solna, Sweden; 3 Science for Life Laboratory, Department of Medicine Solna, Karolinska Institutet, Stockholm, Sweden; 4 The Benaroya Research Institute at Virginia Mason, Seattle, WA USA

**Keywords:** Autoimmunity, Translational immunology, Rheumatoid arthritis, CD4-positive T cells

## Abstract

We aimed to search for common features in the autoreactive T cell receptor (TCR) repertoire in patients with rheumatoid arthritis (RA), focusing on the newly identified candidate antigen citrullinated Tenascin C (cit-TNC). Mononuclear cells from peripheral blood or synovial fluid of eight RA-patients positive for the RA-associated HLA-DRB1*04:01 allele were in-vitro cultured with recently identified citrullinated peptides from Tenascin C. Antigen-specific T cells were isolated using peptide-HLA tetramer staining and subsequently single-cell sequenced for paired alpha/beta TCR analyses by bioinformatic tools. TCRs were re-expressed for further studies of antigen-specificity and T cell responses. Autoreactive T cell lines could be grown out from both peripheral blood and synovial fluid. We demonstrate the feasibility of retrieving true autoreactive TCR sequences by validating antigen-specificity in T cell lines with re-expressed TCRs. One of the Tenascin C peptides, cit-TNC22, gave the most robust T cell responses including biased TCR gene usage patterns. The shared TCR-beta chain signature among the cit-TNC22-specific TCRs was evident in blood and synovial fluid of different patients. The identification of common elements in the autoreactive TCR repertoire gives promise to the possibility of both immune monitoring of the autoimmune components in RA and of future antigen- or TCR-targeted specific intervention in subsets of patients.

## Introduction

MHC/HLA class II-restricted and antigen-specific CD4 + T cells are considered essential in the pathogenesis of ACPA-positive rheumatoid arthritis (RA) and increased knowledge of the specificity and composition of T cell receptors (TCRs) of such T cells will be valuable for the development of novel T-cell directed therapies^1^. We and others have described antigen-specific responses to citrullinated peptides from several potential target molecules^2–4^, including a recent report on T cell reactivity against citrullinated Tenascin C (cit-TNC), where such T cell reactivities were prominent in both peripheral blood (PB) and synovial fluid (SF) of HLA-DRB1*04:01-positive RA patients^5^. TNC is an extracellular matrix protein with increased expression in the rheumatoid joint^6^, and based on description of autoantibodies to its citrullinated form^7^ and the aforementioned study of T cell responses, cit-TNC has become interesting for autoimmunity in RA.

**Figure 1:**
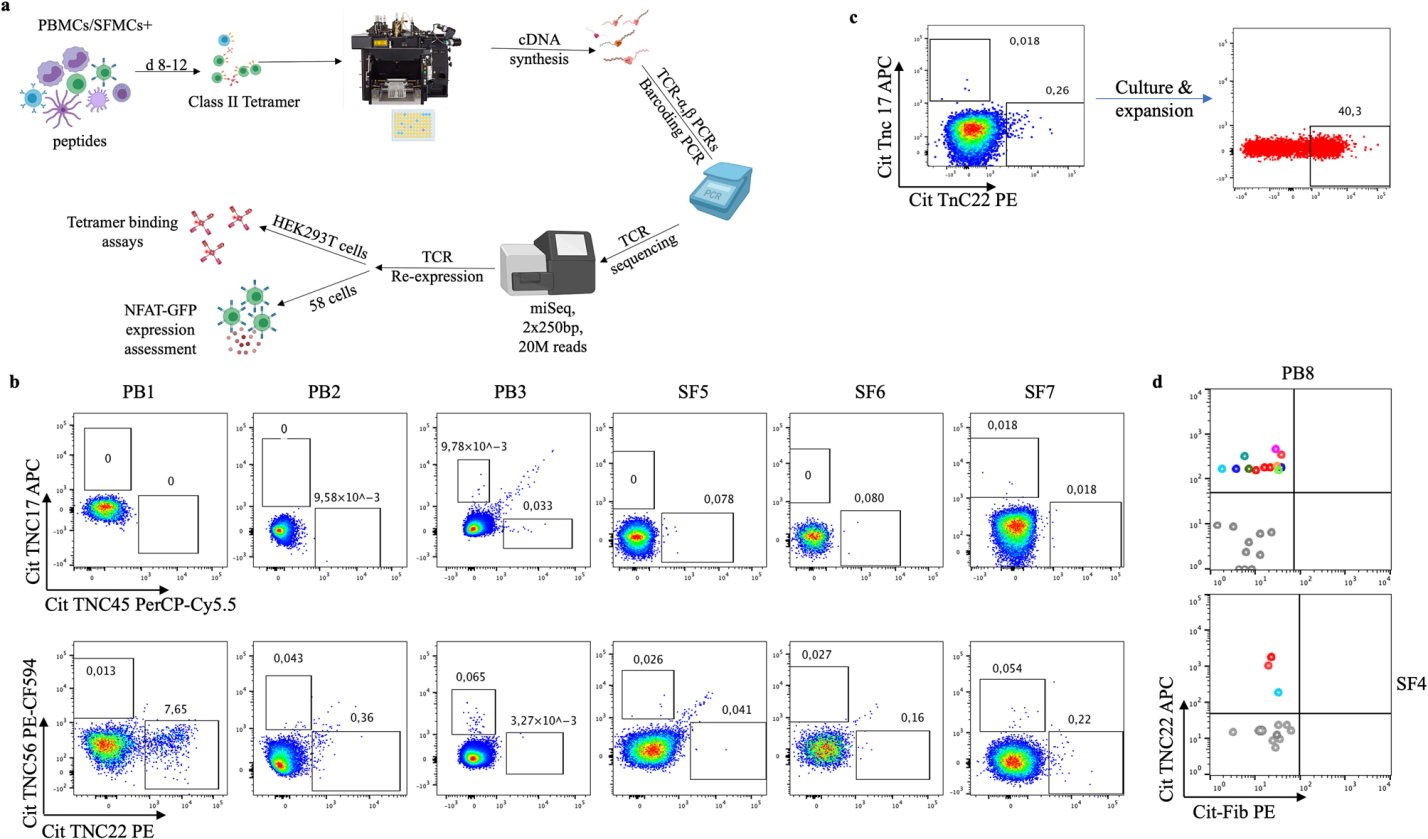
Generation of short-term T cell lines from RA patient material: (**a**) Overview of the TCR discovery pipeline for antigen-specific CD4 + T cells. Patient-derived primary cells are *in-vitro* cultured with cit-TNC peptides or control (influenza) antigens. Antigen-specific T cells are confirmed by peptide-loaded HLA-DRB1*04:01-tetramer staining, single-sorted and processed for cDNA synthesis and PCR amplifications of alpha and beta TCR genes. Barcoded PCR-products are pooled, sequenced and subsequently demultiplexed to obtain individual αβTCRs. Selected TCR sequences are artificially re-expressed either transiently in HEK293T cell line and/or stably in 58αβTCR-negative cell line^10^ for validation of their ability to bind and respond to cognate peptide-MHCII complexes. (**b**) The figure shows screening for an cit-TNC specific response at day 9 of culture in the different peripheral blood (n = 3) and synovial fluid (n = 3) samples. Cit-TNC17, cit-TNC45 (top row) and cit-TNC22, cit-TNC56 (bottom row) specific cells were identified using relevant tetramers. Hereby, Cit-TNC17 + T cells were found in two samples, while 4 of the samples were found to contain cit-TNC45 specific CD4 + T cells (top row). Positive responses to cit-TNC56 was found in three samples, while cit-TNC22 + T cells were found in five samples. (**c**) Tetramer positive cells from one SF culture were bulk sorted and further expanded with PHA. The expanded cells showed extensive enrichment in proportion of cit-TNC22 specific CD4 + T cells. (**d**) This method provides a robust readout to screen immunodominant peptide being presented in an individual and downstream isolation of T-cells specific for that particular epitope. We could exemplify this strategy in one peripheral blood sample (upper panel) and one synovial fluid sample (lower panel), where we used a cocktail of 5 different citrullinated targets (fibrinogen n = 2, enolase n = 1, Tenascin C n = 2). Hereby, we retrieved TCR sequences from bright cit-TNC22 specific CD4 + T cells, both in SF (one expanded TCR present twice, red cells) and PB (two expanded clones, red and blue cells).

Recent developments in single-cell technologies have made TCR repertoire studies easier and more high-throughput^8^. So far, the diversity of the autoreactive TCR repertoire in RA is mostly unknown including whether they share common structural features, something that would facilitate their detection and potential therapeutic targeting. Herein, we have established a pipeline (Fig. 1a) for the isolation and re-expression of paired αβ TCR sequences from RA patient-derived single T cells^9,10^. In this proof-of-principle study utilizing the RA candidate autoantigen citrullinated Tenascin C, we could demonstrate the feasibility of isolation of *in-vitro* expanded autoreactive CD4 + T cells after stimulation with a cocktail of citrullinated peptides, and their subsequent paired alpha–beta TCR sequencing and validation. Moreover, we could identify a biased TCR beta gene usage in response to citrullinated Tenascin C that is shared among patients, indicating structural similarities in the TCR repertoire in RA.

The high abundance and cytokine production capacity^5^, along with the presence of shared TCR features in synovial cit-TNC specific CD4 + T cells from patients with ACPA positive RA suggests an active role for cit-TNC specific immune responses in clinical manifestations and joint damage in this RA subset. This knowledge also motivates future efforts towards design of tolerogenic approaches for restoring immune homeostasis to this extracellular matrix protein.

## Results

### Patient characteristics

Our study included 8 (4 PB, 4SF) HLA-typed patients with HLA-DRB1*04:01 genotype and median age of 52 years. Patients had established disease ranging from 8–43 years duration (median age 17 years). All patients fulfilled ACR/EULAR-2010 RA criteria. Patient characteristics are described in Table 1.

**Table 1:** Patient clinical characteristics.

Feature	n (%)	median (range)
Patients	8 (100)	
Age (years)		52.5 (37–56)
Disease duration (years)		17 (8–43)
Sex F/M	5 (62.5)/3(37.5)	
ACPA-positive	7 (87.5)	
RF-positive	3 (37.5)	
HLA-DRB1*04:01	8 (100)	
ACR/EULAR-2010 fulfillment*	8 (100)	
Current MTX treatment**	4 (50)	
Current anti-TNF treatment	5 (62.5)	
Current glucocorticoid treatment	3 (37.5)	
Current JAK-inhibitor treatment	1(6.25)	
Erosive disease	5 (62.5)	

*One patient was initially diagnosed with JIA but was later re-diagnosed ACPA + RF + rheumatoid arthritis.

**One patient did not have any active antirheumatic treatment.

### Generation of short-term T cell lines from RA patient material

We have recently demonstrated prominent frequencies of cit-TNC specific CD4 + T cells in HLA-DRB1*04:01 patients with RA using ex-vivo HLA tetramer staining and fluorospot assays^5^. Here, we set out to study the TCR diversity of the cit-TNC specific CD4 + T cell response (Fig. 1a). To this end, primary cells from RA patients (Table 1) were in vitro stimulated with four recently identified cit-TNC peptides (Suppl_table 1). The proportion of antigen-specific CD4 + T cells of an individual specificity was assessed using HLA class II tetramers. Upon stimulation of RA-derived primary cells from peripheral blood or synovial fluid of RA patients with the cit-TNC T-cell epitopes, cit-TNC22 was identified as the most common specificity leading to outgrowth of short-term T cell lines (Fig. 1b, suppl_fig. 1). We could validate the specificity of such a cit-TNC22 specific cell line originating from synovial fluid, where cit-TNC22 specific CD4 + T cells after mitogen-driven expansion remained specific for cit-TNC22 peptide HLA-tetramers (Fig. 1c). Notably, these cells were negative for the irrelevant cit-TNC17 HLA tetramer, validating the antigen-specificity of the T cell line. We could also isolate cit-TNC22 specific CD4 + T cells from one PB and one SF samples stimulated with cocktail of 5 different epitopes from 3 different citrullinated protein candidates (Fig. 1d). Collectively, our set-up allows the generation of citrulline-specific T cell lines from RA patient material using in-vitro culture and HLA class II tetramers.

### Cit-TNC specific TCRs can be retrieved and validated for antigen-specificity using artificial re-expression

**Figure 2:**
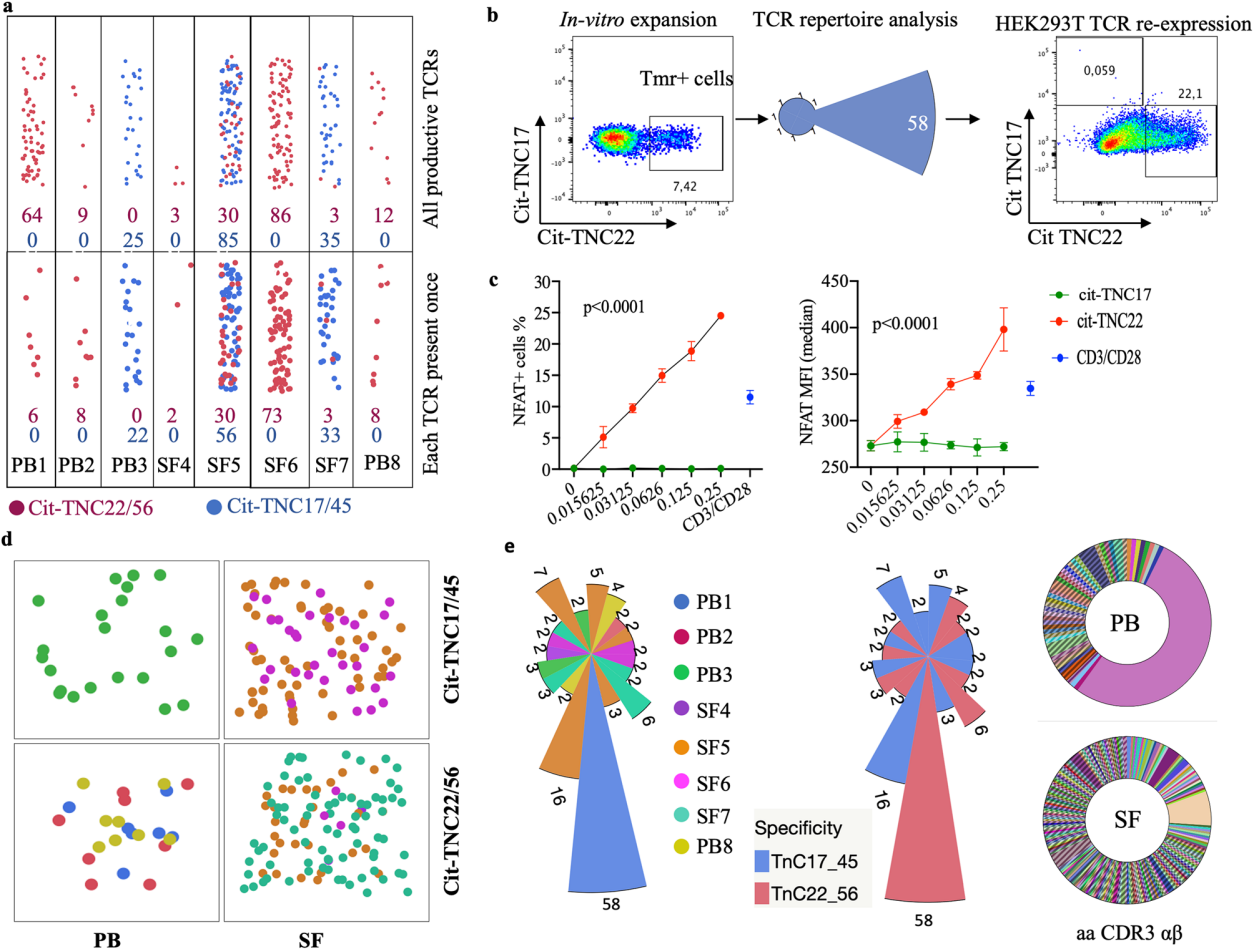
Identification and validation of antigen-specificity of cit-TNC specific TCRs: (**a**) The number of sorted cells that gave productive TCRs after sequencing are shown with cit-TNC22/56 specific cells in burgundy and cit-TNC17/45 specific TCRs in blue color (top panel). Clustering of TCRs using CDR3αβ amino acid sequences showed the exact number of TCRs obtained for the two specificity groups in each patient (bottom panel). Collectively, we could retrieve TCR sequences for cit-TNC17/45 specificity from 3 patients, and cit-TNC22/56 from 7 samples. (**b**) Demonstration of a cit-TNC22 specific CD4 + T cell expansion from peripheral blood. Tetramer staining at day 9 showed an expansion of cit-TNC22 specific CD4 + T cells, comprising one dominant TCR. Transient artificial re-expression of the TCR into HEK293T cells confirmed binding to its cognate tetramer (x-axis), but not to another epitope from cit-TNC17 (y-axis). (**c**) Stable re-expression of same TCR into 58αβ TCR-negative cell line expressing NFAT-GFP reporter demonstrated responsiveness towards cit-TNC22 loaded HLA-DRB1*04:01 monomers, both in proportion of NFAT + cells and of NFAT-GFP median fluorescence intensity (MFI) after stimulation with cit-TNC22 (red symbols) but not with the irrelevant cit-TNC17 peptide-monomer complexes (green symbols). Anti-CD3/CD28 stimulation is shown in blue color. X axis depicts different amounts of peptide-monomer complexes used for coating the plates. (**d**) Figure shows the recovery of TCRs belonging to each of the two specificity groups (cit-TNC17/45 or cit-TNC22/56) from both PB and SF from different patients, where each color represents TCRs derived from a different patient. (**e**) First panel shows TCR repertoire of expanded cit-TNC specific T cells. In total, 19 expanded TCR clones were found in 8 RA patients (4 PB, 4 SF), with varying extent of expansion. Each color in the figure indicates TCRs coming from different patients. Second panel shows all expanded TCRs in context of fine-specificity group (red = cit-TNC22/56 specific, blue = cit-TNC17/45 specific). Among the expanded clones, 9 TCR clones were specific for cit-TNC22/56, while 10 were cit-TNC17/45 specific. Last panel shows the extent of expansion of TCR clones within PB (top) and SF (bottom) compartments, where both sites showed expanded TCR clones.

Studies of autoreactive CD4 + T cells in human disease are hampered by their low frequency and potentially low affinity to their cognate peptide. Hence, it was important to validate the peptide-HLA tetramer results. Here, artificial re-expression of TCRs allows confirmation of antigen-specificity as well as further studies of structural and functional characteristics^10,11^. Thus, we single-cell sorted HLA tetramer-positive T cells for TCR sequencing from our short-term T cell lines (approximately at day 14). From the pool of cit-TNC specific CD + T cells isolated from the different patients, we recovered 352 paired wells showing TCR alpha/beta sequences, all originating from single cells (Fig. 2a, top panel). These TCRs where found to distribute into 246 distinct TCR clones (Fig. 2a, bottom panel). The most striking T cell line originated from a blood sample, constituted almost monoclonal expansion of cit-TNC22 specific T cells, in response to stimulation with the cit-TNC peptide cocktail (Fig. 2b). Artificial transient re-expression of this cit-TNC22 specific TCR in HEK293T cells confirmed its specificity to cit-TNC22 HLA tetramer without any interaction with an irrelevant TNC-peptide (Fig. 2b). Moreover, the same TCR upon permanent re-expression into a stable T-cell line, generated a cit-TNC22-specific immune response with increased NFAT-GFP expression after cognate stimulation with cit-TNC22 HLA-monomeric complexes, again without any response towards another cit-TNC derived (cit-TNC17 HLA-monomers) (Fig. 2c) or unrelated citrullinated fibrinogen derived peptide^11^ (Fibβ-74cit_69-81_) (Supp.Fig. 2) ligands.

### TCR-CDR3 regions show sharing and similarities in amino acid usage between RA patients

Altogether, seven samples provided data for cit-TNC22/TNC56-specific TCRs (Fig. 2a,d,e), and three samples for cit-TNC17/45-specific TCRs (Fig. 2a,d,e). Among PB derived cells, one specimen showed cit-TNC17/45 reactivity while 3 specimens showed cit-TNC22/56 specific cells, (Fig. 2d). Among 246 TCR sequences, 19 total clonal expansions were identified from five different cit-TNC22/56 cultures (3 PB and 2SF, 9 TCR clones) and three cit-TNC17/45 cultures (1 PB and 2SF, 10 TCR clones) (Fig. 2e).

**Figure 3:**
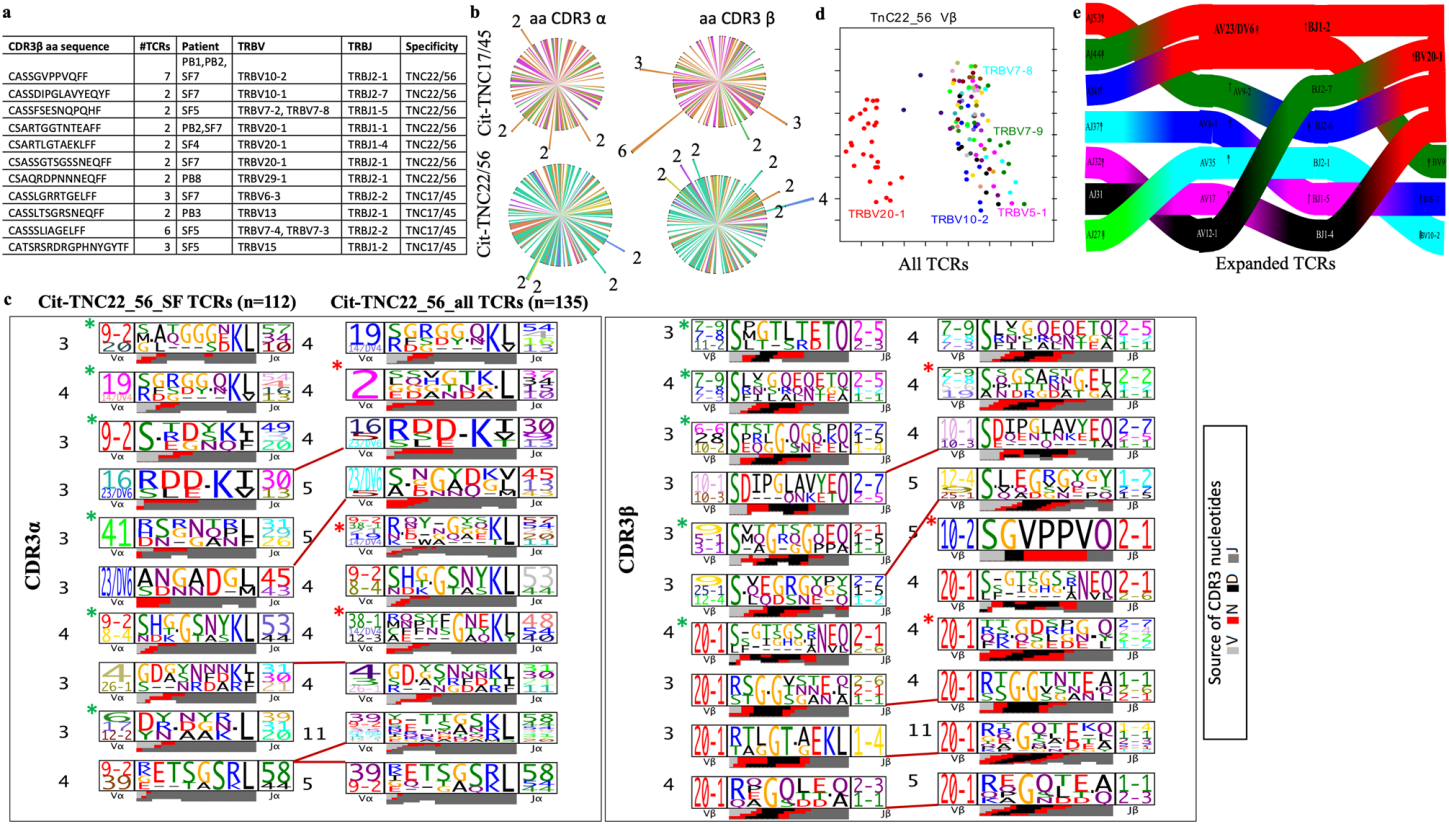
Features of cit-TNC specific TCR repertoire in RA: (**a**) Sharing of amino acid sequences in the TCR-CDR3 beta was found between 3 different patients and within 7/8 patients. Shared TRBV and TRBJ gene usage patterns are shown in the context of the different patient samples and citrullinated antigens. Only cit-TNC22/56 specific CDR3 sequences showed sharing between patients. (**b**) Sharing of exact CDR3α (left column) or CDR3β (right column) amino acid sequences between different TCRs as shown for TNC17/45 sequences (upper row), and TNC22/56 (bottom row). The protruding arms in the figure represent shared TCRs where each color signifies a different sample. (**c**) The figure shows CDR3 consensus sequences (logos) obtained using TCRdist, where amino acids are marked according to their source genes. The first panel shows the comparison of patterns in the CDR3 alpha chain of cit-TNC 22/56 specific repertoire in SF (left column) and all (SF + PB) samples (right column). The second panel shows the patterns in CDR3 beta sequences. For both alpha and beta, the patterns shared between PB and SF of different samples are marked with connecting lines, while SF restricted and PB restricted patterns are marked in green and red asterisks respectively. (**d**) Principal component analysis (PCA) of TRBV gene usage between all cit-TNC22/56-specific TCRs shows differential distribution of TRBV20-1 gene (red) while other gene segments display considerable overlap. (**e**) Paired alpha/beta TCR gene usage patterns of cit-TNC22/56 specific expanded TCRs from the four patients are shown on the right. The gene usage pattern (alpha on the left and beta to the right) show genes in order of their prevalence (top color representing maximum proportion). TRBV20-1 (left side in red) was the most used BV gene and paired gene-usage patterns showed similarities among different cit-TNC22/56-specific TCRs from different RA patients.

Next, we compared different TCRs in terms of their CDR3α and CDR3β regions, which are contributed by junctional nucleotides at V and J (α) or at D and J (β) genes. Although we couldn’t find any public TCRs having identical CDR3αβ amino acid sequences between patients (Fig. 2e), we still observed the same CDR3α or CDR3β between different cit-TNC22/56 specific TCRs present in either same patient or shared between PB and SF of 3 different patients (Fig. 3a,b). Additionally, we found amino acids with similar structural and biochemical properties in CDR3α and CDR3β of different cit-TNC22/56 specific TCRs present in PB and SF of different patients (Fig. 3c). Such a biased TCR repertoire is most likely driven by antigen-specificity.

### Cit-TNC22 specific TCR repertoire shows a distinct bias in TCR beta gene usage

To further test the bias in TCR usage, we compared genes from α or β TCR chains. Several striking features of the cit-TNC22/56 specific TCRs were identified. We found sharing of TCR alpha and beta genes between different sites (Fig. 3c, suppl_fig.3a). Most striking finding was the clustering of TRBV20-1 gene in the overall cit-TNC22/56 specific repertoire, but not in that of cit-TNC17/45 specific repertoire, in patients (Fig. 3d, suppl_fig.3b). Separate analysis of PB and SF derived TCRs also showed over usage of TRBV20-1 for cit-TNC22/56 specific repertoire (suppl_fig.4). TRBV20-1 was the most utilized gene segment both when analyzing all cit-TNC22/56 specific TCRs (present in 28 TCRs from 5 of 7 patients), (suppl_fig.4) as well as TCRs from the expanded clones (Fig. 3e). Such prominent TRBV20-1 bias was not observed amongst influenza-specific T cells (Suppl_fig.3c). Such similarities in TCR usages further strengthens the idea of antigen driven changes in TCR repertoire.

## Discussion

RA represents a disease with clear HLA-DR association^12^, distinct autoimmune features^13^, and an increasingly long list of candidate autoantigens^1–5,14,15^. Here we have focused on recently identified T-cell epitopes from citrullinated Tenascin C, to which T cells have been found to be prevalent, in some cases in close parity to that of virus-specific (influenza) T cells^5^. Our findings of shared features in the RA TCR repertoires are both conceptual, demonstrating feasibility for additional studies with other candidate autoantigens, and also have direct implications for continued efforts to monitor this autoreactivity in patient samples and even as a basis to design specific interventions.

In RA, both peripheral blood and the inflamed joint are relatively accessible from a research perspective. Still, the scarcity of autoreactive T cells continues to make studies challenging. Our method of *in-vitro* stimulating patient-derived primary cells with proposed autoantigens in presence of recombinant human IL-2 and IL-7 allows the propagation and subsequent capture of these rare cells for downstream studies of the T cell receptor repertoire^16^, where IL-7 was especially important for the synovial cultures. Importantly, the method allows for stimulation with either peptides (single or cocktails) or proteins^10^ and can also be useful for T cell epitope mapping studies.

Altogether, several findings suggest citrullinated Tenascin C to be a relevant autoantigen in RA. Apart from the relatively high frequencies of cit-TNC T cells^5^, making this autoreactivity suitable for our proof of principle approach from the pathogenesis perspective; native Tenascin C is an extra cellular matrix (ECM) protein which is virtually absent from the circulation of healthy individuals but in contrast, it is upregulated in the context of inflammation, such as in the rheumatic joint^6,17^. In patients with RA, native TNC can also be found in the circulation and associated with erosive disease^17,18^. Citrullinated tenascin C has hence been indirectly implicated as an inflammation dependent ACPA target^7^ but we eagerly anticipate more studies focused on the modified version of this protein and its distribution.

Utilizing peptide-HLA multimer technology, antigen-specific CD4 + T cells have been demonstrated in ‘classical’ autoimmune diseases as type 1 diabetes and SLE, and also in celiac disease, Parkinson’s disease and atherosclerosis^8,19–23^. In some disease settings, the presence of antigen-specific T cells have been associated to disease progression^8,22^. Despite these advancements, the αβTCR-repertoire of autoreactive CD4 + T cells in many autoimmune diseases including rheumatoid arthritis has remained less understood, with the exception of celiac disease^24^.

Interestingly, we could see a similarity in amino acid usage in CDR3 regions of different TCRs from CD4 + T cells in our cohort of established disease patients. This sharing of patterns (i.e. CDR3 which are crucial for epitope recognition in T cells), between anatomical sites and among different patients strongly suggests the existence of structural similarities between cit-TNC22 specific TCRs. Additionally, we observed several CDR3 motifs sharing TRBV20-1 in citrullinated TNC22 specific TCRs in different RA patients, all having HLA-DRB1*04:01 genotype. Biased usage of TCR gene segments have been demonstrated for example, a preference for TRBV5 in CD4 T cells specific for a hybrid insulin epitope has been reported^25^. Similarly, TCR alpha chain usage (TRAV26-1) was shown to be over-represented in context of gluten epitope in celiac disease^26^. Our observed bias in TRBV-usage is suggestive of a similar structural recognition mechanism.

Admittedly, our cohort is small, but this fact also underscores our data since we find shared TCR features between sites in different patients. Moreover, all the patients in our cohort have long standing RA (range 8–43 years), and the presence of autoreactive T cells in all samples suggests that the patients have either long-lived memory T cells or are continuously exposed to citrullinated Tenascin C. Having knowledge on which TCR genes or segments are overused in context of autoimmunity, can potentially be translated into targeting of these specific segments in order to re-introduce immune homeostasis. An essential pre-requisite for achieving this ambition is to ensure that those elements are not crucial for generating immune responses to common pathogens. Our observation that influenza specific repertoire didn’t show overuse of TRBV20-1 gives a preliminary model to design such studies. Our data implicate that in addition to introduction of tolerance using antigens, an alternative approach can be to perturb the immune receptor repertoire by deleting some specific TCRs. Here, the generation of TCR-transgenic or retrogenic mice^10^ would facilitate investigations of both potential pathogenicity of TCRs and the potentials to re-regulate T cell responses by tolerizing approaches.

To summarize, we demonstrate the usefulness of technologies that enable the identification and sequencing of large numbers of antigen-specific/autoreactive TCRs, followed by their functional validation. We exemplify this technology in the context of citrullinated Tenascin C and demonstrate shared features between cit-TNC-specific T cells from different HLA-DRB1*04:01-positive RA patients and between PB and SF. All our patients had established disease and we hope our data will motivate similar studies also for other candidate RA autoantigens and ultimately investigations on autoreactive T cells and TCR repertoires at the RA at-risk phase, in early disease and in the context of disease progression and therapeutic intervention.

## Materials and methods

### Patient material

Research samples from RA patients (n = 8) carrying at least one allele of HLA-DRB1*04:01 were included in this study. Mononuclear cells from peripheral blood (PB, n = 4) and synovial fluid (SF, n = 4) were prepared by ficoll separation and cryopreserved until use. The study was approved by the institutional ethics committee at Karolinska Institutet. Informed consent was obtained from the patients, prior to sample collection. Patient characteristics are summarized in Table 1. All experiments were performed in accordance with relevant guidelines.

### In-vitro culture and tetramer staining

Mononuclear cells from PB and SF were cultured in presence of cit-TNC peptide cocktail (suppl_table.1) or influenza matrix protein MP_97-116_ (in separate wells) containing each peptide at a concentration of 10 ug/mL in complete RPMI1640 containing 10% human serum (Sigma Aldrich). Cells were also stimulated with rhIL-2 at day 6 followed by addition of IL-2 every 2 days at a concentration of 10 IU/mL. Assessment of reactivity to specific cit-TNC peptide and screening for expansion of antigen-specific CD4 + T cells was performed using 4 different peptide-loaded HLA-DRB1*04:01 tetramers (citTNC17-APC, citTNC22-PE, citTNC45-PerCP-Cy5.5 and citTNC56-PE-CF594). Influenza reactivity was assessed in separate tubes using MP_97-116_ PE tetramers. Briefly, an aliquot of cells was taken around day 9 from each culture and stained with anti-human CD3-BV786, CD4-BV510, CD14-APC-H7, CD16-APC-H7 and CD19-APC-H7 antibodies (1:100, BD Biosciences). Cells were then washed with 1X PBS and stained with tetramers (1:100) and incubated at 37 °C for 60 min. Cells were then washed and stained with near-IR fixable viability dye (1:1500) (Thermofisher Scientific) by incubating for 10 min in dark, washed twice and acquired on flow cytometer (BD LSR Fortessa). Viable C3 + CD4 + singlet T cells were identified as shown (suppl_fig 1). We considered the presence of 3 or more bright cells (more than one log difference between negative and positive population) to be indicative of a response. Cultures were harvested at day 14, tetramer staining was done using a cocktail of tetramers in 2 color combinations (cit-TNC17 APC, cit-TNC45 APC and cit-TNC22 PE, cit-TNC56 PE) and tetramer positive cells were single-sorted in indexed manner using flow cytometry (BD influx). One well in each sorted plate was left blank as a negative control, while 5 CD4 + T cells each in 3 wells were sorted as positive control for TCR sequencing.

### TCR sequencing and data analysis

Antigen-specific CD4 + T cells were single sorted and processed for TCR sequencing using a previously published approach^9^. Briefly, cDNA synthesis was followed by two rounds of PCRs to amplify TCR alpha/beta genes, followed by barcoding of products to combine multiple antigen-specific TCRs. The sequencing data was demultiplexed using custom in-house script and TCR alignment was done using mixcr software. Clones with more than 20 reads each for α and β chain genes were selected for further analysis^24^. The TCRs were analyzed for clonal expansion on the basis of comparison of CDR3 amino acid sequence from combined α and β chains. TCR data was analyzed using JMP15 and TCRdist. TCRs were analyzed for differences in context of amino acid and chain usage using TCRdist^27^. The tool makes logos on the basis of biochemical and structural similarities among different amino acids being used at a particular position. The TCR logos have residue height scaled according to their frequency in the repertoire, while the colored bars underneath represent the source of the nucleotides that encode the CDR3 region. The tool considers all clonally expanding TCRs to be one, so as to avoid bias from over-representation of one or few TCRs.

### TCR re-expression and validation

Cit-TNC22 specific expanded TCR was re-expressed either transiently in HEK293T (stably expressing mouse CD3 and human CD4) cell line or stably in 58TCRαβ-negative cell line using a recently published approach^10^. Transient re-expression was used for assessment of binding of TCR to its cognate peptide-HLA complex. Stable TCR re-expressing cell line was used to assess the impact of TCR stimulation via peptide-HLA complex on the expression of reporter (NFAT-GFP). 48 well plates were coated with different amounts of monomeric HLA-peptide complexes (in PBS) for 4 h and 5 × 10^4^ cells were added to each well. Monomeric peptide-HLA complexes were coated on 48-well plates starting from 0.25ug in serial dilution, while anti-mouse CD3 antibody was coated at a concentration of 1ug/mL. Cit-TNC17 was used as a negative control. 5 × 10^4^ cells were added to each well and cells were incubated at 37 °C and 5% CO_2_ for 48 h and assessed for NFAT-GFP expression using flow cytometry^10^. Experiments were repeated 3 times and data shown is median ± interquartile range in the graphs.

## Supplementary Information

Supplementary Information.

## Data Availability

Raw data can be provided on a written request to the corresponding author.
