# Supplementary material for: Biased TCR gene usage in citrullinated Tenascin C specific T-cells in rheumatoid arthritis
Source: Sci Rep. 2021 Dec 31;11:24512. doi: 10.1038/s41598-021-04291-8 (PMC8720095; doi:10.1038/s41598-021-04291-8)
Supplement: Supplementary file 1 — Supplementary Information. [file 41598_2021_4291_MOESM1_ESM.pdf]

Biased TCR gene usage in citrullinated Tenascin C specific T-cells in Rheumatoid arthritis

Ravi K. Sharma[1,2], Sanjay V. Boddul[1,2], Niyaz Yoosuf[1,2], Sara Turcinov[1,2], Anatoly Dubnovitsky[1,3], Genadiy Kozhukh[1,3], Fredrik Wermeling[1,2], William W. Kwok[4], Lars Klareskog[1,2], Vivianne Malmström[1,2,*]

[1]Department of Medicine, Rheumatology Unit, Karolinska Institutet, Karolinska University Hospital, 171 76 Stockholm, Sweden

[2]Center for Molecular Medicine, Karolinska Institutet, Solna, Sweden

[3]Science for Life Laboratory, Department of Medicine Solna, Karolinska Institutet, Stockholm, Sweden

[4]The Benaroya Research Institute at Virginia Mason, Seattle, WA, USA

*Corresponding author

Prof. Vivianne Malmström,

Division of Rheumatology,

Department of Medicine,

Karolinska Institutet, Solna, Sweden

e-mail: vivianne.malmstrom@ki.se

ORCID: 0000-0001-9251-8082

Supp.Table 1: cit-TNC peptides used in the study:

S.No	Epitope	Sequence	Cit location/peptide length
1.	Cit-TNC17	[H]VSLIS[X][X]GDMSSNPA[OH]	TNC-876-877-X (871-885)
2.	Cit-TNC22	[H]FD[X]Y[X]LNYSLPTGQW[OH]	TNC-1014-1016-X (1012-1026)
3.	Cit-TNC45	[H]PDGF[X]LSWTADEGVF[OH]	TNC-1637-X (1633-1647)
4.	Cit-TNC56	[H]QGQYEL[X]VDL[X]DHGE[OH]	TNC-2073-2077-X (2067-2081)

X-citrulline

Supplementary figures:

1. Suppl_fig.1. Gating strategy for selecting tetramer positive cells:

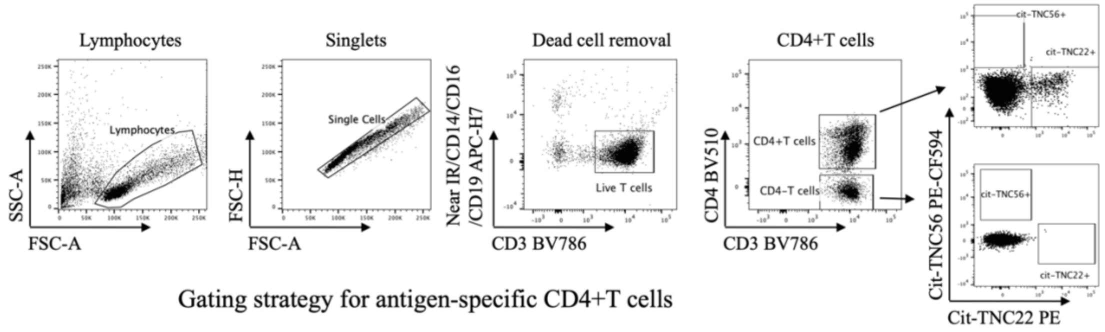

Representative gating strategy to characterize antigen-specific CD4+T cells is demonstrated. Lymphocytes were gated on the basis of forward and side scatter, followed by gating of singlets and discrimination of dead cells, monocytes, NK cells and B cells. CD3+T cells were then selected as being either CD3+CD4+ (test population) or CD3+CD4- (control population). The extent of tetramer staining on CD3+CD4+T cells (upper panel) and absence of tetramer positive events on CD3+CD4- T cells (lower panel) demonstrates the specificity of the staining.

Suppl_fig.2: Specificity of cit-TNC22 specific TCR:

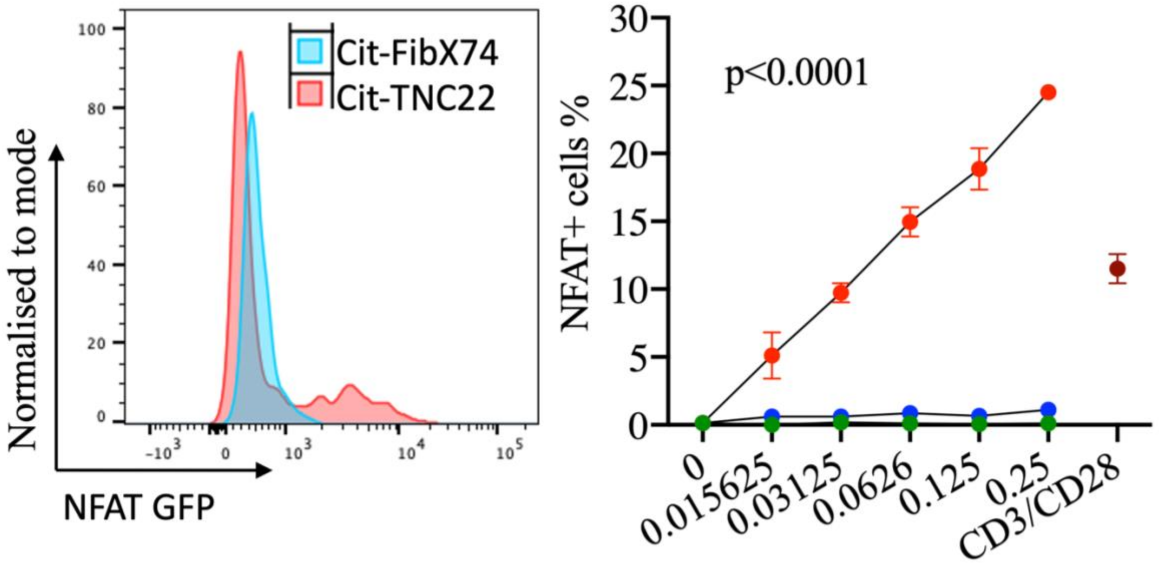

To validate the specificity of cit-TN22 specific TCRs, we tested the response of 58 cell line towards Fibβ-74cit$_{69\text{-}81}$ (an epitope on Fibrinogen β) complexed with HLA-DRB1*04:01 monomers. First figure shows the expression of NFAT-GFP on 58 cell lines after stimulation with Fibβ-74cit$_{69\text{-}81}$ (aqua color) overlaid by cit-TNC22 (red color) stimulation. The second figure (extension of fig.2c) shows lack of response to NFAT activation in 58 cells stimulated with Fibβ-74cit$_{69\text{-}81}$ (blue color) and cit-TNC17 (green color) but a positive response with cit-TNC22 (red color) and CD3/CD28 (brown color) stimulation. X axis represents different amounts of peptide-monomer complexes used in the experiment, while Y axis represents frequency of NFAT expressing cells.

Suppl_fig.3: Gene sharing patterns in citrullinated Tenascin C specific TCR repertoire in RA:

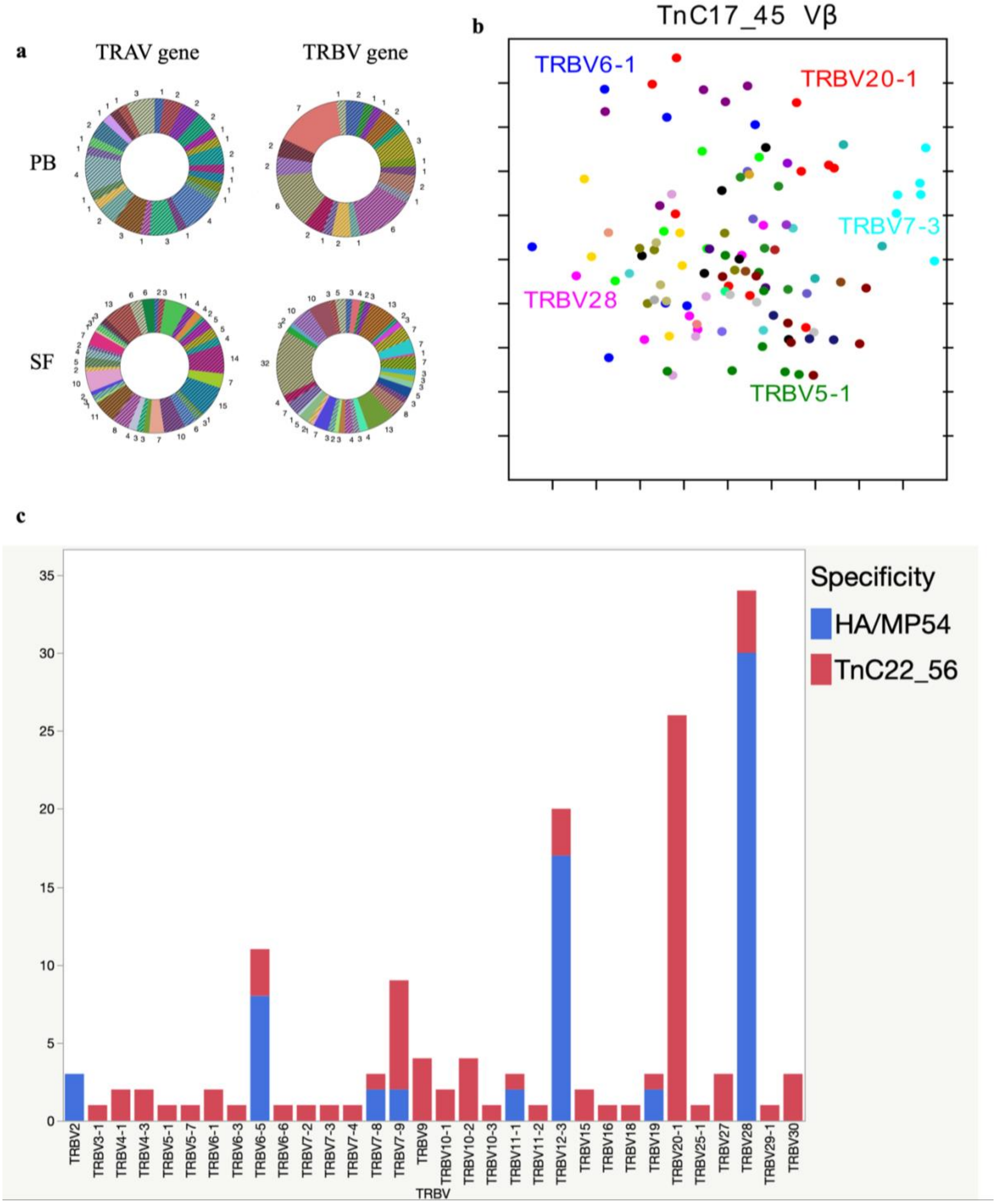

(A) The left panel shows the extent of expansion and overall size of TCR repertoire in PB (n=3) and SF (n=4). Expanded clones are marked with the numbers corresponding to the size of the expansion. No clones were identical between PB and SF of RA patients. The middle panel

shows TRAV gene usage in all cit-TNC22/56 specific TCRs in PB and SF, with shared genes in the pie chart marked with striped colors. The right panel shows gene sharing for TRBV genes in all cit-TNC22/56 specific TCRs from the two compartments, again with shared genes in the pie chart marked with lines. (B) Principal component analysis (PCA) of TRBV gene usage between all cit-TNC17/45 specific TCRs performed using TCRdist shows some bias in gene usage, but different to that of TNC22/56 specific TCRs. (C) The figure shows data from one patient where we sequenced both cit-TNC22/56 and influenza MP97 specific TCRs from synovial fluid. We found over-representation of TRBV20-1 in cit-TNC22/56 specific cells but not in influenza specific TCRs, further validating the biased gene usage pattern observed from different comparisons.

Suppl_fig.4. Similarities in gene usage patterns between PB and SF:

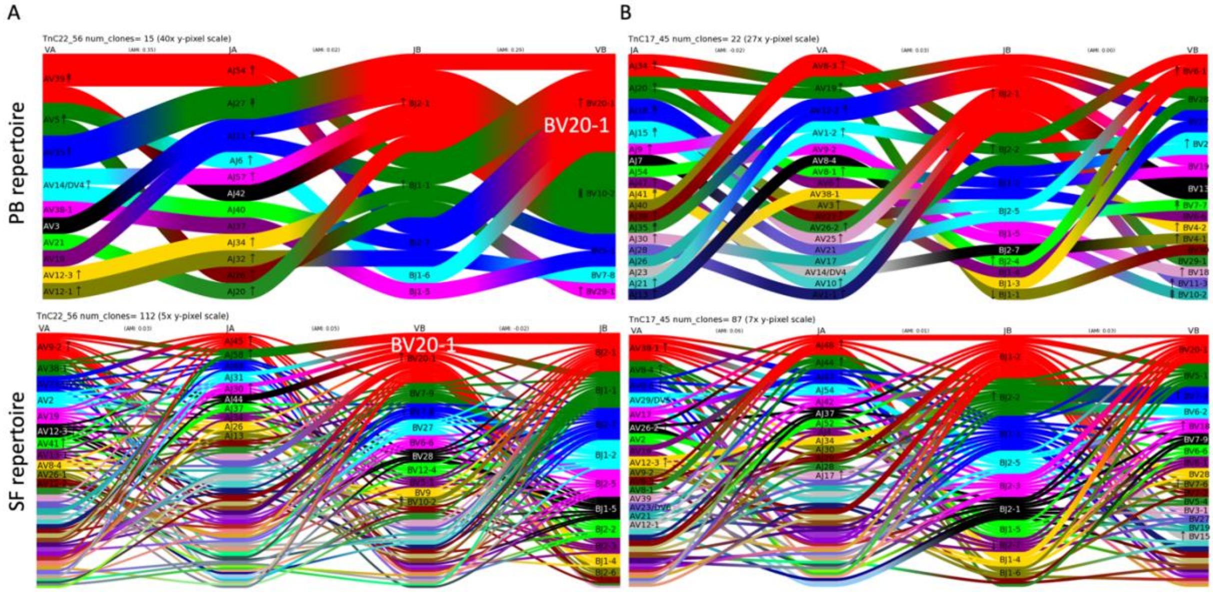

TCRdist was used to plot gene-gene pairing landscapes to display the patterns of paired alpha and beta genes in TCRs of the two different specificities in both PB (upper panel) and SF (lower panel). Each color represents a distinct clonotype and the thickness of the line is proportional to the frequency of the clonotype. The figure A clearly shows similarities in TRBJ (TRBJ2-1) gene usage and over re-presentation of TRBV20-1 in cit-TNC22/56 specific TCRs in both compartments. Figure B shows paired gene usage for cit-TNC17/45 specific TCRs, with differential TRBV gene usage in PB and different combination of TRBV-TRBJ in SF, as compared to cit-TNC22/56 specific TCRs. TRBJ2-1 and TRBJ1-1 were most preferentially used genes in cit-TNC22/56 specific repertoire in both sites while the corresponding genes in SF of cit-TNC17/45 specific TCRs were TRBJ1-2 and TRBJ2-2.
